# Screening for the ciliate *Buxtonella sulcata* in free-ranging dairy cattle on Terceira Island, Azores archipelago

**DOI:** 10.1051/parasite/2025014

**Published:** 2025-03-28

**Authors:** Mário Ribeiro, Sara Gomes-Gonçalves, Alexandra Silva, Guilherme Moreira, Eric Viscogliosi, Magali Chabé, João Rodrigo Mesquita

**Affiliations:** 1 Microbiology and Infectious Diseases Laboratory (MIDlab), ICBAS – School of Medicine and Biomedical Sciences, Porto University, Rua de Jorge Viterbo Ferreira 228 Porto Portugal; 2 Univ. Lille, CNRS, Inserm, CHU Lille, Institut Pasteur de Lille, U1019 – UMR 9017 – CIIL – Centre d’Infection et d’Immunité de Lille 59000 Lille France

**Keywords:** *Buxtonella sulcata*, Dairy cattle, Polymerase chain reaction, Protist

## Abstract

*Buxtonella sulcata* is an alveolate ciliate protist, historically considered a commensal of the gastrointestinal tract of cattle. Despite the fact that its cysts are morphologically identical to those of *Balantioides coli*, molecular identification techniques have shed new light on its role as a pathogen. This work aimed to assess the presence of this ciliate in the population of dairy cattle on the Azorean island of Terceira by means of molecular analyses (ITS1–5.8S–ITS2 rRNA) of stool samples. A total of 116 samples were collected from adult Holstein-Friesian dairy cows, with no signs of gastrointestinal disease. A proportion of 49.1% of the samples were PCR-positive for *Bu. sulcata*, and 12 different genetic sequences were identified. These findings highlight the need for future research concerning the factors that influence the presence of *Bu. sulcata* in the gastrointestinal tract of dairy cows, the role of bovines as possible sources of infection, and the impact this ciliate may have on the health, welfare, and productivity of these animals.

## Abstract (fr)

*Buxtonella sulcata* est un protiste cilié alvéolé, historiquement considéré comme un commensal du tube digestif des bovins. En dépit du fait que la morphologie de ses kystes est identique à celle de *Balantioides coli*, les techniques d’identification moléculaire ont apporté un nouvel éclairage sur son rôle comme pathogène. Ce travail vise à évaluer la présence de ce cilié dans la population de vaches laitières de l’île de Terceira, aux Açores, grâce à des analyses moléculaires (ARNr ITS1–5.8S–ITS2) d’échantillons de selles. Au total, 116 échantillons ont été prélevés sur des vaches laitières Holstein adultes, ne présentant aucun signe de maladie gastro-intestinale. Une proportion de 49,1 % des échantillons étaient positifs à la PCR pour *Bu. sulcata* et 12 séquences génétiques différentes ont été identifiées. Ces résultats soulignent la nécessité de recherches futures concernant les facteurs qui influencent la présence de *Bu. sulcata* dans le tractus gastro-intestinal des vaches laitières, le rôle des bovins comme sources possibles d’infection et l’impact que ce cilié peut avoir sur la santé, le bien-être et la productivité de ces animaux.

## Introduction

*Buxtonella sulcata*, an alveolate protist of the phylum Ciliophora (ciliates), was first described by Jameson in 1926 [1]. It has similarity to other members of this phylum (which includes *Balantioides coli*), and *Bu. sulcata* has long been considered to be a commensal organism. It is one of many ciliates that populate the gastrointestinal (GI) tract of cattle [1], where certain ciliates assist in breaking down cellulose. Consequently, and given that the cysts of *Ba. coli* are almost identical to those of *Bu. sulcata* [15], the formerly named *Balantidium coli* (now *Balantioides coli*) has long been recognised as the only ciliate of veterinary and human medical interest, associated with GI disease [1]. However, with the increasingly widespread use of molecular identification and DNA sequencing, *Bu. sulcata* has been detected more frequently, and its potential role as a pathogen and primary cause of diarrhoea in bovine populations has recently been proposed [8]. This, in turn, has led to increased awareness of its potential impact on animal productivity and the associated economic losses. Consequently, it has become necessary to understand the extent to which this ciliate is present in apparently healthy bovine populations, so as to determine the potential role of *Bu. sulcata* carriers as silent shedders. To the best of our knowledge, no data currently exist for the bovine populations of the Azorean archipelago, a region of Portugal where dairy cattle managed under an intensive rotational grazing system abound. Therefore, our study aimed at helping to bridge this knowledge gap by analysing stools from non-diarrhoeic bovines of the Azorean island of Terceira.

## Material and methods

### Ethics

The experiments described in this paper required vertebrate faecal matter as a source and did not subject animals to any additional interventions, invasive or otherwise. The samples were collected in the course of routine fertility checks performed by the veterinarians who were kind enough to assist the researchers. These checks require trans-rectal palpations and ultrasonographic examinations, which, in turn, require the emptying of the rectal ampulla of the cows under examination and, by extension, the removal of faeces. A portion of these faeces, which would otherwise have been discarded, was collected for research purposes. Given that no activities were conducted besides those that would have been performed by the large animal clinicians regardless of the existence of this paper, no ethics committee approval was required.

### Sample location and feeding system

Terceira Island (Azores, Portugal) is divided into two municipalities, Angra do Heroísmo (AH) and Praia da Vitória (PV). Similarly to the rest of the Azorean archipelago, the island features a characteristically oceanic climate, with mild temperatures (17 °C, ranging from 9 to 26 °C, and small differences between the summer and winter months) and an annual rainfall of 900 to 3000 mm [14]. Over 90% of the designated agricultural area of Terceira Island is employed as pasture for cattle (almost exclusively Holstein-Friesian), with average herd sizes of 41.1 cows [19].

Their pasture-based diet, mostly composed of perennial ryegrass (*Lolium perenne*) and white clover (*Trifolium repens*), was occasionally supplemented with commercial feed (the primary constituents of which were corn and soybean) and forage (mostly corn silage).

### Sampling

**Figure 1:**
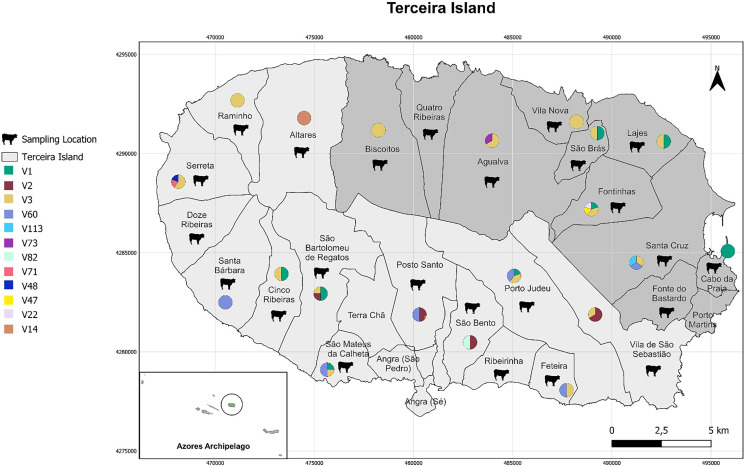
Description of the sampling used in this study (depicted as black cows), along with a graphical representation of the relative frequency with which the different *Buxtonella sulcata* sequences were identified in each respective parish. The pie charts display these sequence frequencies with specific colour labels.

A total of 116 bovine faecal samples were collected from animals undergoing routine fertility checks, with 70 samples from Angra do Heroísmo and the remaining 46 from Praia da Vitória. Table 1 provides a detailed description of the sampling locations by municipality and civil parish, and Figure 1 illustrates the geographic position of these sampling sites.

**Table 1:** Distribution of sampling locations by municipality and civil parish of Terceira Island.

Municipality	Civil parish	Number of samples (*n*)
Angra do Heroísmo	Altares	1
	Cinco Ribeiras	6
	Doze Ribeiras	2
	Feteira	10
	Porto Judeu	7
	Posto Santo	4
	Raminho	5
	Ribeirinha	1
	Santa Bárbara	3
	São Bartolomeu dos Regatos	7
	São Bento	5
	São Mateus da Calheta	5
	Serreta	7
	Vila de São Sebastião	7
Praia da Vitória	Agualva	5
	Biscoitos	2
	Cabo da Praia	2
	Fonte do Bastardo	2
	Fontinhas	9
	Lajes	6
	Quatro Ribeiras	2
	Santa Cruz	7
	São Brás	6
	Vila Nova	5

Stool samples were collected from adult female Holstein-Friesian dairy cows, all of which were maintained under a permanent intensive rotational grazing system. The collection period extended from October to December 2023. All cows were assessed as healthy by the attending veterinarians, with a body condition score between 2 (thin) and 3 (moderate), according to the body condition scoring system for dairy cows [17], and a normal rectal temperature, with a range from 38.0 °C to 39.3 °C [13].

The samples were collected either directly from the rectum of the cows following transrectal palpation or immediately after defecation. All of the samples exhibited a consistency score of either 0 (normal consistency) or 1 (semi-formed or pasty), according to the faecal consistency scoring system [10]. None of the cows exhibited diarrhoea at the time of sampling. The samples were rapidly stored at 4 °C, maintained at this temperature during transportation to the laboratory for a maximum of 8 h, and then preserved at −20 °C until DNA extraction.

### *DNA extraction and molecular detection of* Buxtonella sulcata

Genomic DNA was isolated from approximately 200 mg of all stool samples using a NucleoSpin 96 Soil kit (Macherey-Nagel GmbH & Co KG, Düren, Germany), adhering to the manufacturer’s guidelines. The DNA was eluted in 100 μL of the provided elution buffer and stored at −20 °C until further analysis.

The detection of *B. sulcata* was carried out using a conventional PCR method targeting the ITS1–5.8S–ITS2 rRNA region with the B5D/B5RC primers. These primers amplify 530 bp fragments, which were subsequently trimmed to approximately 400 bp for alignment [16]. PCR reactions, with a total reaction mix of 25 μL consisting of 5 μL of template DNA and 0.4 μM of each primer and RNAse free water, were conducted with a T100 thermocycler (Bio-Rad, Hercules, CA, USA) using the Speedy Supreme NZYTaq 2× Green Master Mix (NZYTech, Lisbon, Portugal). The PCR protocol consisted of an initial denaturation at 95 °C for 5 min, followed by 40 cycles of denaturation at 94 °C for 2 s, annealing at 60 °C for 5 s, and extension at 72 °C for 5 s, with a final extension at 72 °C for 10 min.

After amplification, the DNA fragments were separated on 1% agarose gels stained with Xpert Green Safe DNA gel dye (GRiSP^®^, Porto, Portugal). Electrophoresis was performed at a constant voltage of 120 V for 30 min, DNA bands were observed under UV light.

### Sanger sequencing and phylogenetic analysis

For Sanger sequencing, all 57 PCR amplicons of the expected size were purified using a GRS PCR & Gel Band Purification Kit (GRiSP^®^, Porto, Portugal). Sequencing was then conducted on both strands with the primers used for PCR. The resulting sequences were edited, aligned, and analysed using BioEdit Sequence Alignment Editor, version 7.2.5. Consensus sequences from each isolate were compared with those in the NCBI GenBank database through the nucleotide basic local alignment search tool (BLAST). A total of 20 homologous *Buxtonella* sequences were selected based on availability, with preference given to those from similar hosts to ensure biological relevance and minimise host-related divergence. Phylogenetic tree reconstruction was carried out using MEGA X software, which selected the Tamura-3-Parameter model as the most appropriate for the analysis. To assess statistical reliability, bootstrap values were computed using 1000 replicates with the chosen model. A discrete Gamma distribution was applied to account for evolutionary rate variations among sites, with partial deletion of sites containing gaps exceeding 5%. The alignment length was 400 bp.

The sequences obtained in this study have been deposited in GenBank (PQ002351 to PQ002407).

### Statistical analysis

The occurrence of *Bu. sulcata* in bovine faecal samples was determined by calculating the proportion of positive samples to the total number of samples analysed, with a 95% confidence interval (95% CI).

## Results

*Buxtonella sulcata* molecular detection methods performed on the 116 bovine faecal samples revealed that 49.1% (57/116; 95% CI: 39.7–58.6) were PCR-positive with an expected amplicon size of 530 bp. Sequencing and subsequent BLAST analysis confirmed the detection of *Bu. sulcata* in all positive PCR samples.

A total of 12 different *Bu. sulcata* sequences were found in this study. The sequences V1 (PQ002407), V2 (PQ002406), V3 (PQ002405), and V60 (PQ002380) served as representatives for multiple genetically *Bu. sulcata* identical sequences found in this work, as indicated in Table 2. These representative sequences were used to emphasise unique evolutionary relationships and improve the clarity of the phylogenetic tree. The other *Bu. sulcata* sequences (V113, V73, V82, V71, V48, V47, V22, and V14) were only detected in individual samples. Figure 1 illustrates the geographic representation of the sampling sites and the various *Bu. sulcata* sequences identified in each respective parish.

**Table 2:** Distribution of *Buxtonella sulcata* categorical sequences across various parishes. The table presents the percentages of different *Bu. sulcata* sequence categories (V1, V2, V3, V60, Singular) for each parish, along with the total number of positive samples. Percentages reflect the proportion of each sequence group (category) within each parish.

Municipality	Civil parish	V1 (%)	V2 (%)	V3 (%)	V60 (%)	Singular (%)	Total
Angra do Heroísmo	Altares	0	0	0	0	100	1
	Cinco Ribeiras	66.7	0	33.3	0	0	3
	Feteira	0	0	50	50	0	4
	Porto Judeu	20	0	40	40	0	5
	Posto Santo	0	50	0	50	0	2
	Raminho	0	0	100	0	0	4
	Santa Bárbara	0	0	0	100	0	1
	São Bartolomeu dos Regatos	50	25	25	0	0	4
	São Bento	0	50	0	0	50	2
	São Mateus da Calheta	25	0	25	50	0	4
	Serreta	0	0	60	0	40	5
	Vila de São Sebastião	0	66.7	33.3	0	0	3
Praia da Vitória	Agualva	0	0	66.7	0	33.3	3
	Biscoitos	0	0	100	0	0	1
	Fontinhas	20	0	40	0	40	5
	Lajes	75	0	25	0	0	4
	Santa Cruz	0	0	33.3	33.3	33.3	3
	São Brás	100	0	0	0	0	2
	Vila Nova	0	0	100	0	0	1

Table 2 summarises the distribution of *Bu. sulcata* sequences in the various parishes. It details the percentage representation of each sequence category – V1, V2, V3, V60 or other – in each civil parish, as well as the total number of positive samples, providing an overview of how different sequences are distributed geographically.

Sequence group V3 (PQ002405) was detected relatively uniformly throughout the island, excluding the civil parishes of Altares (AH), Santa Bárbara (AH), Posto Santo (AH), São Bento (AH), and São Brás (PV), and it was the only sequence identified in the northern/northwestern regions of Raminho (AH), Biscoitos (PV), and Vila Nova (PV). In contrast, sequence group V60 (PQ002380) was present only in southern/southeastern civil parishes, namely Santa Bárbara (AH), São Mateus da Calheta (AH), Posto Santo (AH), Porto Judeu (AH), Feteira (AH), and Santa Cruz (PV). It was the only sequence identified in Santa Bárbara (AH). Sequence group V1 (PQ002407) displayed a less uniform pattern of distribution, being detected in the southwestern civil parishes of Cinco Ribeiras (AH), São Bartolomeu de Regatos (AH), and São Mateus da Calheta (AH), in the southern civil parish of Porto Judeu (AH), and in the northeastern civil parishes of Fontinhas (PV), Lajes (PV), and São Brás (PV), being the only detected sequence in the latter. Lastly, sequence group V2 (PQ002406) was only detected in four of the southern civil parishes of the island [São Bartolomeu de Regatos (AH), Posto Santo (AH), São Bento (AH), and Vila de São Sebastião (AH)]. The remaining singular sequences were only found in one civil parish each: sequence V113 in Santa Cruz (PV), sequence V73 in Agualva (PV), sequence V82 in São Bento (AH), both sequences V71 and V48 in Serreta (AH), sequence V47 and V22 in Fontinhas (PV), and sequence V14 in Altares (AH). Within the same civil parish, all of the singular sequences were identified alongside one or more of the sequence groups V1, V2, V3, and V60, with the exception of sequence V14, which was the only one detected in Altares (AH).

**Figure 2:**
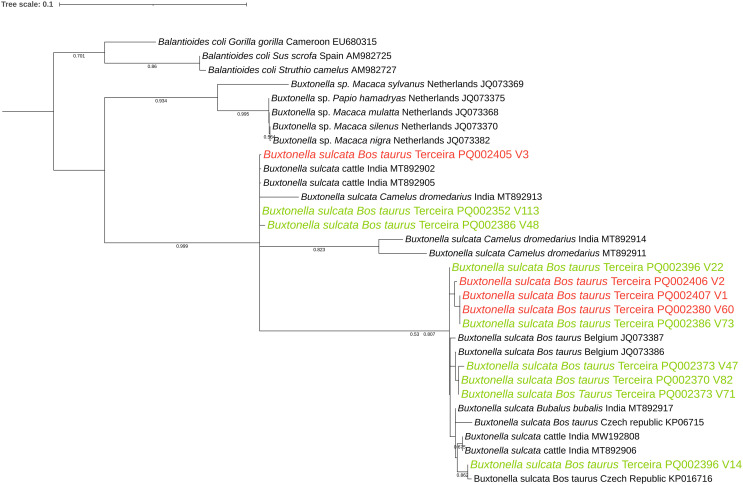
Phylogenetic analysis of *Buxtonella sulcata* sequences obtained in this study and reference genotypes, identified with respective accession numbers, host, and country of origin. The phylogenetic tree was performed using the highest likelihood method and Tamura 3-parameter model. A discrete Gamma distribution was used to model evolutionary rate differences among sites, using partial deletion of sites with gaps exceeding 5%. Red and green entries indicate sequences obtained in this study, with the red entries representing multiple identical sequences.

Table 3 displays the genetically identical *Buxtonella sulcata* sequences, which have been grouped into four representative categories: V3, V60, V1, and V2. BLASTN analysis of sequences V1(PQ002407), V22 (PQ002396), V47 (PQ002387), V48 (PQ002386), V71 (PQ002373), V73 (PQ002368), and V113 (PQ002352) showed the highest match with *Bu. sulcata* sequence JQ073387 (cattle, Belgium), with identities ranging from 98.8% to 99.76%. Sequences V3 (PQ002405) and V60 (PQ002380) matched *Bu. sulcata* sequence MT892905 (cattle, India) with identities of 100% and 99.76%, respectively. Sequence V14 (PQ002351) displayed a 99.52% match with MT892917 (water buffalo, India). Sequences V2 (PQ002406) and V82 (PQ002370) matched JQ073386 (cattle, Belgium) with identities of 99.76% and 99.52%, respectively. Phylogenetic analysis of the resulting amplicons confirmed that the *Bu. sulcata* sequences obtained in our study grouped with those from cattle (Fig. 2).

**Figure 3:**
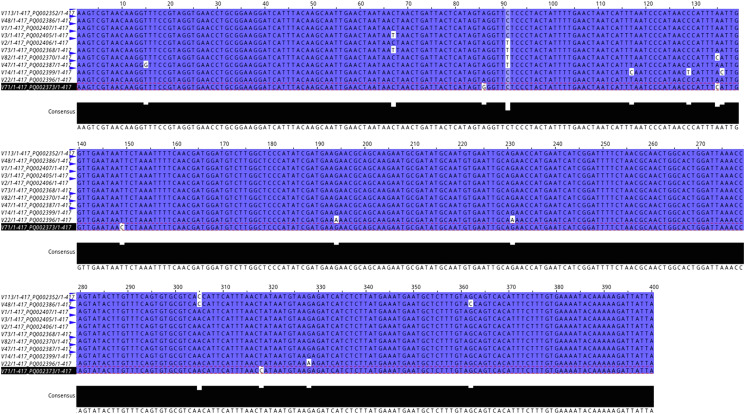
Alignment of *Buxtonella sulcata* ITS sequences obtained in this study.

**Table 3:** Identification of genetically identical sequences represented by V3, V60, V1, and V2.

V3 (PQ002405)	V4 (PQ002404); V5 (PQ002403), V7 (PQ002402), V15 (PQ002398), V21 (PQ002397), V31(PQ002393), V33 (PQ002391), V43 (PQ002389), V53(PQ002385), V59 (PQ002381), V63 (PQ002379), V67 (PQ002376), V69 (PQ002375), V70 (PQ002374), V72 (PQ002372), V78 (PQ002371), V93 (PQ002364), V98 (PQ002361), V101 (PQ002359), V102 (PQ002358), V109 (PQ002356), V110 (PQ002355), V111 (PQ002354), V112 (PQ002353)
V60 (PQ002380)	V58 (PQ002382), V65 (PQ002378), V66 (PQ002377), V87 (PQ002367), V97 (PQ002362), V99 (PQ002360), V103 (PQ002357), V114 (PQ002351)
V1 (PQ002407)	V57 (PQ002383), V56 (PQ002384), V34 (PQ002390), V32 (PQ002392), V45 (PQ002388), V25 (PQ002395), V27 (PQ002394), V9 (PQ002400), V8 (PQ002401)
V2 (PQ002406)	V96 (PQ002363), V91(PQ002365), V84 (PQ002369), V90 (PQ002366)

## Discussion

Determining the role of ciliates in GI disease can be challenging. The GI tract of herbivores, in particular, hosts a multitude of different ciliates which seem to be neither pathogenic nor indispensable to their hosts [1]. Furthermore, the interactions between the GI tract and a specific protist population can vary significantly from host to host. For example, swine are considered to be the principal hosts and major reservoirs of *Balantioides coli*, suffering no harmful effects as a result of the presence of this parasite [15]. Further confusing matters, many of these protists proliferate considerably during episodes of diarrhoea, despite having no aetiological importance [1].

*Buxtonella sulcata* has been classically considered one of these apparent commensals [1]. Nevertheless, this ciliate has also been reported as a cause of GI disease [3, 8, 9], with the incidence of diarrhoea increasing proportionally with the intensity of infection [4]. In addition to being implicated in coinfections with other parasites, such as *Eimeria* spp. and *Toxocara vitulorum*, *Bu. sulcata* has been more frequently detected in cases of diarrhoea, particularly in instances of single infection. This suggests its potential role as a primary pathogen in GI disease [3].

The 116 cows screened in this study were all adults. Although there have been reports to the contrary [6], it has been suggested that the prevalence of infection by *Bu. sulcata* (and the negative consequences thereof) is higher in calves than in adults [9]. In fact, some researchers consider *Bu. sulcata* a vital pathogenic element and the most common cause of diarrhoea in calves [8]. Age has been proposed as one of the major risk factors in the spread of parasitic infection, with morbidity and risk of infection being greater in younger animals [3] due to their immature immune system. Additionally, changes in the GI microenvironment, such as pH fluctuations as a result of feeding, may favour the multiplication of *Bu. sulcata* [2]. In light of this, it may be possible to expand the group of individuals most at risk to include those animals that are immunocompromised or in a pro-inflammatory state as a result of comorbidities. Concerning the GI microenvironment, the greater variety of different microorganisms that are a part of the normal intestinal microflora of adult bovines could also help to control the activity of potentially pathogenic agents. All of the samples from this study originated from non-diarrhoeic Holstein-Friesian cows that were deemed healthy by the attending veterinarians. It would have been interesting to include calf faeces in the sample pool. Further studies should aim at investigating both the parasitic populations of the GI tract of calves from the Terceira Island and of adult bovines that had presented with diarrhoea as calves.

Given its presumed nature as a commensal and its geographically disperse detection in the bovine population under study, one might suppose that *Bu. sulcata* features somewhat ubiquitously in the GI tracts of cattle populations, similar to other unicellular commensals, and may even potentially contribute in some manner to the maintenance of normal physiological processes [11]. Interestingly, one survey, in which researchers subjected 166 bovine stool samples from seven farms in mainland Portugal (from Setúbal, Évora and Santarém) to molecular identification methods, did not yield positive results for *Bu. sulcata* or *Ba. coli* by employing the same primer set as in the present study [5].

This discrepancy in the GI microenvironment between bovine populations of relatively close regions could be the result of several factors. The climate of the Azorean archipelago is notably more temperate and humid [14] compared to the central and southern regions of Portugal where [5] collected their samples. Additionally, the feeding conditions of dairy cows from the mainland are also distinct from those found in the Azores, with the latter group of animals being much more dependent on pasture. The Azorean cows in the present study were part of extensive farming systems, while [5] included samples from both extensive (*n* = 24) and intensive (*n* = 142) farming systems. Other aspects that could influence the presence of this ciliate include the type of feeding (and specifically, the type of pasture) to which the animals have access, along with the associated contamination risks. Moreover, only 49.1% of the samples in the present study tested positive for *Bu. sulcata*, suggesting that individual (host) factors significantly influence the occurrence of this ciliate in the bovine GI tract. All of these variables warrant greater investigation.

As evidenced above, sequence groups V60 and V2 appear to be restricted to the southernmost regions of the island, while sequence group V3 features more uniformly throughout the territory. The distribution of the different sequence groups and their relationships with singular sequences might also present an interesting topic for future research.

Although the trophozoites of *Bu. sulcata* and *Ba. coli* can be differentiated through careful microscopic examination, their cysts are almost indistinguishable [15]. Historically, *Ba. coli* has been regarded as the only ciliate of significant concern in both veterinary and human medicine [1]. However, the occurrence of this parasite in several species, including primates, has mostly been reported in microscopy-based studies [12]. Since the molecular identification of these ciliates has started to be routinely applied relatively recently, the relevance that has so far been attributed to *Bu. sulcata* in the overall landscape of potentially pathogenic protists may now be called into question. It is noteworthy that *Buxtonella*-like species have been identified through DNA sequencing in both captive and wild primate species, including hamadryas baboons (*Papio hamadryas*), rhesus macaques (*Macaca mulatta*) and mangabeys (*Cercocebus torquatus*) [15, 18]. These findings suggest potential transmission of *Buxtonella* sp. to humans, whether directly from other animal hosts or through environmental interactions such as water contamination.

In any event, these interesting hypotheses require further study. Since the vast majority of investigative efforts have been directed at *Ba. coli*, a greater body of work concerning the molecular identification of *Bu. sulcata* is warranted. Studies of this type would enhance our understanding of its impact on both human and animal health and welfare. Knowledge of its presence in seemingly healthy bovine populations arms both veterinary and human medical clinicians with important epidemiological information; specifically, regarding possible sources of infection. Additionally, given the wide range of species in which *Buxtonella*-like have been detected and the significance of other ciliates in medical contexts [7], future research should also explore the role of *Buxtonella* species as GI pathogens in other domestic animals, such as, for example, horses and dogs. This would help to further elucidate its potential zoonotic risks and impact on diverse animal populations.

## Conclusions

Unlike previous studies surveying cattle populations within the Portuguese territory, which did not detect *Bu. sulcata*, 49.1% of the faecal samples from dairy cows on the Azorean Island of Terceira tested positive for this ciliate. Notably, none of the animals exhibited signs of GI disease. This discrepancy underscores the need for further research to understand the complex factors influencing the presence of *Bu. sulcata* in the GI tract of ruminants and its potential role as a disease agent in adult bovines. In particular, future studies should evaluate the occurrence of diarrhoea in calves from Terceira Island and examine the resulting parasitic populations in their GI tracts once they reach adulthood. Investigating the role of dairy cows as possible sources of infection, as well as the impact of this ciliate on animal welfare and productivity, is crucial. Employing molecular techniques to detect and study this previously overlooked parasite will be essential in advancing our knowledge and addressing these concerns.
